# Activated full-length myosin-X moves processively on filopodia with large steps toward diverse two-dimensional directions

**DOI:** 10.1038/srep44237

**Published:** 2017-03-13

**Authors:** Osamu Sato, Hyun Suk Jung, Satoshi Komatsu, Yoshikazu Tsukasaki, Tomonobu M. Watanabe, Kazuaki Homma, Mitsuo Ikebe

**Affiliations:** 1 Department of Cellular and Molecular Biology, University of Texas Health Science Center at Tyler, 11937 US HWY 271, Tyler, Texas 75708, USA; 2 Department of Biochemistry, College of Natural Sciences, Kangwon National University, 1, Kangwondaehak-gil, Chuncheon-si, Gangwon-do 24341, Korea; 3 Department of Pharmacology, University of Illinois College of Medicine, 808 S Wood St., Chicago, Illinois 60612, USA; 4 Laboratory for Comprehensive Bioimaging, RIKEN Quantitative Biology Center, 6-2-3, Furuedai, Suita, Osaka 565-0874, Japan; 5 Department of Otolaryngology-Head and Neck Surgery, Feinberg School of Medicine, Knowles Hearing Center, Northwestern University, 320 E Superior, Chicago, Illinois 60611, USA

## Abstract

Myosin-X, (Myo 10), is an unconventional myosin that transports the specific cargos to filopodial tips, and is associated with the mechanism underlying filopodia formation and extension. To clarify the innate motor characteristic, we studied the single molecule movement of a full-length myosin-X construct with leucine zipper at the C-terminal end of the tail (M10^Full^LZ) and the tail-truncated myosin-X without artificial dimerization motif (BAP-M10^1–979^HMM). M10^Full^LZ localizes at the tip of filopodia like myosin-X full-length (M10^Full^). M10^Full^LZ moves on actin filaments in the presence of PI(3,4,5)P_3_, an activator of myosin-X. Single molecule motility analysis revealed that the step sizes of both M10^Full^LZ and BAP-M10^1–979^HMM are widely distributed on single actin filaments that is consistent with electron microscopy observation. M10^Full^LZ moves on filopodial actin bundles of cells with a mean step size (~36 nm), similar to the step size on single actin filaments (~38 nm). Cartesian plot analysis revealed that M10^Full^LZ meandered on filopodial actin bundles to both x- and y- directions. These results suggest that the lever-arm of full-length myosin-X is flexible enough to processively steps on different actin filaments within the actin bundles of filopodia. This characteristic of myosin-X may facilitate actin filament convergence for filopodia production.

Myosin-X is a member of the myosin superfamily and found in vertebrates and in filasterea^1^,^2^. Myosin-X exhibits a striking localization at the tips of filopodia^1^,^3^,^4^,^5^,^6^,^7^ which suggests that myosin-X moves towards the filopodial tips. Moreover, myosin-X overexpression leads to a dramatic increase in the number and length of filopodia^1^, while knockdown of the expression of endogenous myosin-X by small interference RNA led to the loss of filopodia^4^,^5^. These findings suggest that myosin-X plays a critical role in filopodia formation.

Myosin-X is composed of a conserved motor domain in its N-terminal region, a neck region consisting of three IQ motifs that serve as light chain binding sites, a predicted coiled-coil domain and a unique tail domain^8^. It is now known that myosin-X dimerizes via an antiparallel coiled-coil motif at the distal region of the predicted coiled-coil domain^9^. The tail contains a PEST domain, three pleckstrin homology domains, a myosin tail homology 4 (MyTH4) domain and a band 4.1/ezrin/radixin/moesin (FERM) domain^10^.

The tail domain was reported to bind to and transport specific cargo molecules, such as VASP and ß-integrins^3^,^11^, although the transportation of ß-integrins are not directly shown. Therefore it was assumed that myosin-X is a processive motor which is suitable for cargo transporter. Supporting this view, it was found that myosin-X is a high duty ratio motor^12^. Processive movement of myosin-X with exogenous parallel forced dimer motifs on various actin structures has been controversial. The tail-truncated myosin-X with exogenous forced dimerization motif at the C-terminal end of the endogenous coiled-coil can processively move on fascin-actin bundles but is less processive on single actin filaments^13^,^14^. On the other hand, Sun *et al*.^15^ showed that the tail-truncated forced dimer of myosin-X moves on single actin filaments as well as actin bundles without notable difference in processivity. The reason for the difference is still unclear, although Vavra *et al*. claim that these constructs could be structurally different^16^. The step size of myosin-X is also controversial. Ricca and Rock^17^ showed that the step size of the tail-truncated forced dimer of myosin-X (1–920 amino acids) is 18 nm on both actin filaments and bundles, while Sun *et al*.^15^ reported that the tail-truncated forced dimer of myosin-X (1–939 amino acids) moves on actin bundles predominantly with 34 nm steps and minor 18 nm steps while the step size is 34 nm on single actin filaments. Bao *et al*. revealed that the tail-truncated forced dimer of myosin-X (1–940 amino acids) takes ~31 nm steps in both single and actin bundles^14^. Takagi *et al*.^18^ recently reported that tail-truncated forced dimer of myosin-X (1–936 amino acids) takes 35 nm forward steps on single actin filaments in single molecule optical tweezers experiments. One of the key clues to solve the contradiction may be the dimerization of myosin-X at distal coiled-coil region which is reported to make anti-parallel coiled-coil^9^.

A critical finding is that full-length myosin-X is a monomer having a folded conformation, in which the tail domain folds back to attach the motor-neck domain, although it has a coiled-coil domain between the IQ domain and the PEST domain^19^. This finding indicates that the coiled-coil of myosin-X is not strong enough to produce a stable dimer, which is consistent with the dissociation constant (*K*d) of myosin-X dimer, ∼0.6 μM^9^. A question is how myosin-X can be a cargo transporting motor if it is a monomeric myosin. Answering this question, it was found that phosphatidylinositol 3,4,5-trisphosphate (PI(3,4,5)P_3_) binding at the PH domain of the tail activates the motor activity and induces dimer formation^19^. These results suggest that full-length myosin-X becomes active upon binding PI(3,4,5)P_3_ and that the endogenous coiled-coil domain can form dimer although the stability of the dimer is weak.

It was found previously that an active myosin-X dimer without the cargo binding tail can induce actin filament convergence at the cell’s leading edge to initiate filopodia formation^5^. However, produced filopodia is very short and unstable, and it is thought that the tail domain is important for producing long and stable filopodia. The tail domain can interact with actin regulatory proteins such as VASP and β-integrin, and it is plausible that these actin-interacting proteins are involved in the myosin-X induced filopodia formation process^3^,^11^. Alternatively, the full-length myosin-X dimer might be more effective at converging actin filaments than the artificially forced dimer. These findings suggest that it is important to study the motor activity of the full-length myosin-X to clarify the innate motor characteristic of myosin-X.

In this study, we attempted to clarify the motor properties of full-length myosin-X. To facilitate the dimer formation at the endogenous coiled-coil domain, we introduced a leucine zipper at the C-terminal end of the tail. We also produced and studied a myosin-X construct without artificial coiled-coil to ensure the formation of dimer with innate coiled-coil. Both constructs moved processively on actin filaments with broad step size distribution with major step of 34–38 nm.

## Results

### Processive movement of M10^Full^LZ on single actin filaments

**Figure 1:**
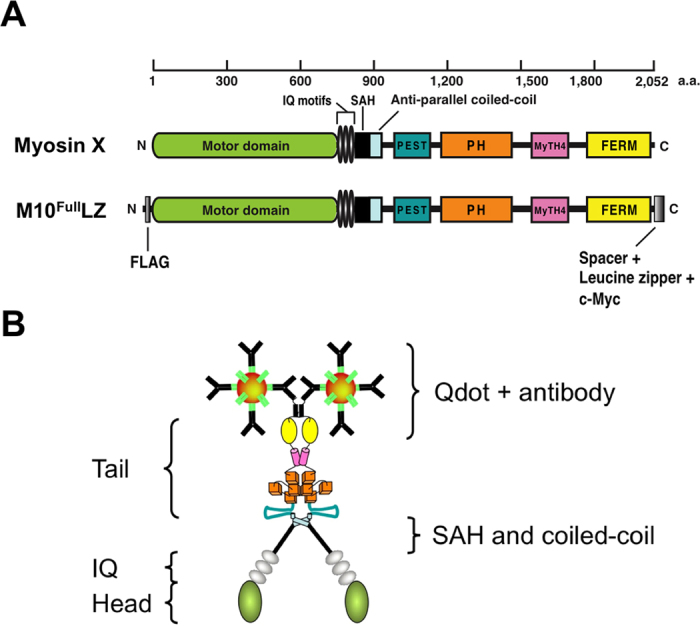
Schematic drawing of the myosin-X construct used in this study. (**A**) Cartoon of domain structures of full-length myosin-X construct. Myosin-X consists of motor domain, 3 IQ motifs, and stable α-helix, coiled-coil domain, and tail containing PEST, PH, MyTH4, and FERM domains. To help single molecule assay, a GCN4 leucine zipper motif and c-Myc sequences were introduced at the C-terminal end of myosin-X. (**B**) Configuration of M10^Full^LZ-Qdot. The Qdots were attached to the C-terminal c-Myc tag of M10^Full^LZ via 1^st^ (anti-mouse Fab’) and 2^nd^ (anti-c-Myc) antibodies.

We constructed recombinant bovine full-length myosin-X containing 1–2052 native myosin-X heavy chain (Fig. 1A, Supplementary Fig. 1A). We placed a GCN4 leucine zipper motif at the C-terminal end of the tail domain to combine the two heavy chains at the C-terminal end, thus facilitating the dimerization at the endogenous coiled-coil domain of myosin-X. We subcloned this recombinant myosin-X construct both in baculovirus and mammalian cell expression vectors. It has been known that myosin-X induces filopodia and localizes at the filopodial tips^4^,^5^. To check the functional authenticity, we examined whether this myosin-X construct facilitates filopodia formation in mammalian cells (Supplementary Fig. 2). The expression of the M10^Full^LZ in COS7 cells notably increases the number of filopodia and M10^Full^LZ showed distinct localization at the tip of filopodia, suggesting that M10^Full^LZ has an authentic function of myosin-X. To prepare the M10^Full^LZ protein, Sf9 cells were co-infected with the myosin-X heavy-chain-expressing virus with calmodulin-expressing virus. To activate M10^Full^LZ, PI(3,4,5)P_3_ diC8 was mixed with M10^Full^LZ. The multi-molecule *in vitro* motility assay showed that purified M10^Full^LZ moves Rhodamine-labeled actin filaments in PI(3,4,5)P_3_ dependent manner, similarly to how M10^Full^ does^19^. Qdot was attached to the C-terminal end of an isolated M10^Full^LZ through anti-c-Myc antibodies as shown in Fig. 1B. From Poisson distribution, the ratio of M10^Full^LZ: Qdot of 1: 20 assures that ~97% of moving Qdot molecules have M10^Full^LZ single molecule.

**Figure 2:**
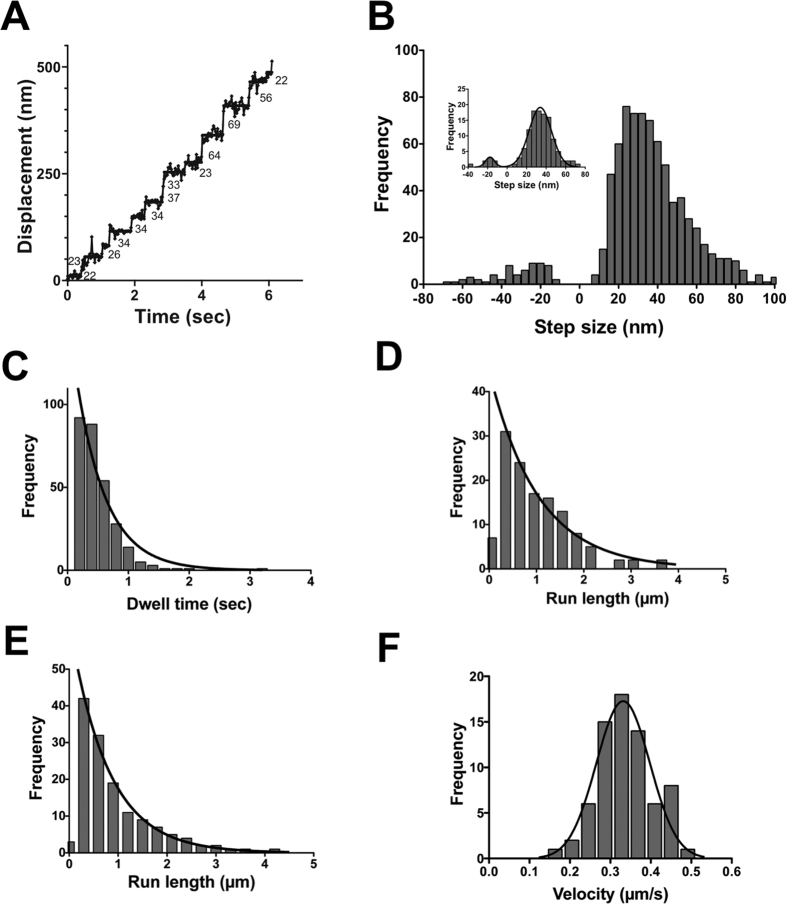
Processive movement of full-length myosin-X dimer (M10^Full^LZ) on single actin filaments at both low and high ATP concentrations. (**A**) A representative trace of M10^Full^LZ stepping. Experiments were done by using M10^Full^LZ-Qdot525 in the presence of 2 μM ATP, and the fluorescence images were captured at 33.3 fps. Solid line represents the best fit to the trajectory. The numbers in the panel are the displacement of each step. (**B**) Step size distribution of M10^Full^LZ in the presence of 2 μM ATP. The mean step size of forward and backward steps are 38.2 ± 17.5 nm (mean ± s.d., n = 731) and −31.4 ± 14.4 nm (s.d., n = 62), respectively. The inset shows the step size distribution of M10^1–939^M5CC. The mean step size of forward and backward steps are 35.1 ± 12.3 nm (mean ± s.d., n = 112) and −19.9 ± 8.0 nm (s.d., n = 8), respectively. Black solid line shows the best fit to Gaussian equation. (**C**) Dwell time distribution of M10^Full^LZ in the presence of 2 μM ATP. Solid line shows the best fit to a single exponential equation, *k*e^−*kt*^, where *t* is time, and *k* is rate constant. The average waiting time (*τ* = 1/*k*) is 0.49 ± 0.05 s (s.e.m., n = 313). (**D**) Run length of M10^Full^LZ in the presence of 2 μM ATP. Solid line shows the best fit to a single exponential equation, *R*_*0*_e^−*r/λ*^, where *R*_*0*_ is the initial frequency extrapolated to zero run length, *r* is run length, and *λ* is average run length (the first bin is excluded from the fitting). The average run length was 1.04 ± 0.08 μm (s.e.m., n = 127). (**E**) Run length of M10^Full^LZ in the presence of 2 mM ATP. The fluorescence images were captured at 4 fps. Solid line shows the best fit to the single exponential equation as described in **D** (the first bin is excluded from the fitting). The average run length was 0.78 ± 0.03 μm (s.e.m., n = 139). (**F**) The velocity of M10^Full^LZ in presence of 2 mM ATP. The best fit to Gaussian equation gives the mean velocity of 0.33 ± 0.01 (s.e.m., n = 71).

We first observed the successive continuous movement of M10^Full^LZ on single actin filaments in the presence of 2 μM ATP using a TIRF microscope. M10^Full^LZ processively moves along single actin filaments as previously reported with the tail-truncated forced dimer of a myosin-X construct, in which the exogenous coiled-coil was introduced immediately after the endogenous coiled-coil domain^15^,^17^. By tracking the center position of myosin-X-Qdots using FIONA technique^20^, we determined the step sizes of M10^Full^LZ (Fig. 2A and B). The step size distribution of M10^Full^LZ was asymmetrical wide distribution with a notably long step size, in contrast to myosin Va HMM labeled with Qdots at the C-terminus, which showed a symmetrical step size distribution without long step size (Supplementary Fig. 3). The mean step size of M10^Full^LZ was 38.2 ± 17.5 nm (mean ± s.d., N_steps/Qdots_ = 731/59) for forward step and −31.4 ± 14.4 nm (s.d., N_steps/Qdots_ = 62/34) for backward step. The mean forward step was slightly longer than a half pitch of F-actin helix (~36 nm). It should be noted that back step was only single steps and we did not observe successive back steps. The obtained forward step size distribution was wide, and best explained with two different step sizes, i.e., 29.1 ± 10.9 nm (s.d.) and 55.3 ± 11.0 nm using the sum of two Gaussian distribution (R^2^ = 0.973). The former step size is consistent with the step sizes previously obtained for the tail-truncated forced dimer constructs^15^,^17^. The latter step size is close to 3/4 pitch of actin filaments. Since >50 nm steps are not seen in M10^1–939^M5CC^15^ (Fig. 2B, inset) or myosin Va HMM (Supplementary Fig. 3), the latter step is considered to be unique to M10^Full^LZ. Note that while some >70 nm steps can be considered missing steps below the resolution of the measurement (estimated to be 3–6% in total steps), this cannot explain the observed 20–30% of the ~50 nm steps of M10^Full^ LZ.

Figure 2C is dwell time distribution of M10^Full^LZ at 2 μM ATP. The average waiting time is 0.49 ± 0.05 s (s.e.m., 0.45 ± 0.01 s from Kaplan-Meier survival curve, see also Supplementary Fig. 5Aa), which is consistent with the M10^1–939^M5CC value (0.89 s at 1 μM MgATP^15^). The average waiting time gives the single turnover rate of ~2 s^−1^ at 2 μM ATP, which is 1/10–1/2 value of the *V*_max_ of myosin-X (4–20 s^−1^)^12^,^19^,^21^. Run length distribution of M10^Full^LZ gave the average run length of 1.04 ± 0.08 μm (s.e.m.) at 2 μM ATP (Fig. 2D), which is similar to M10^1–939^M5CC (~1 μm^15^), but much longer than M10^1–920^LZ (~0.2 μm^13^,^22^). To test whether M10^Full^LZ moves processively on single actin filaments in physiological ATP concentration, we observed the movement at 2 mM ATP (Fig. 2E and F). M10^Full^LZ also processively moves at 2 mM ATP with average run length of 0.78 ± 0.03 μm (s.e.m.), slightly shorter than that in 2 μM ATP. This indicates that M10^Full^LZ moves 20–25 steps on average at the physiological concentration of ATP on single actin filaments. The velocity of the movement at 2 mM ATP is 0.33 ± 0.01 μm/s. This means that M10^Full^LZ takes 10 steps per second, i.e., ~10 s^−1^, which is much larger than the result of *V*_*max*_ value of steady-state actin-activated ATPase activity of full-length myosin-X reported previously^19^, but similar to the *V*_*max*_ of myosin-X S1^12^. This may be because the motility assay measures only activated myosin-X and it is known that full-length myosin-X activity is regulated^19^.

### Processive movement of M10^1–979^ without LZ on single actin filaments

**Figure 3:**
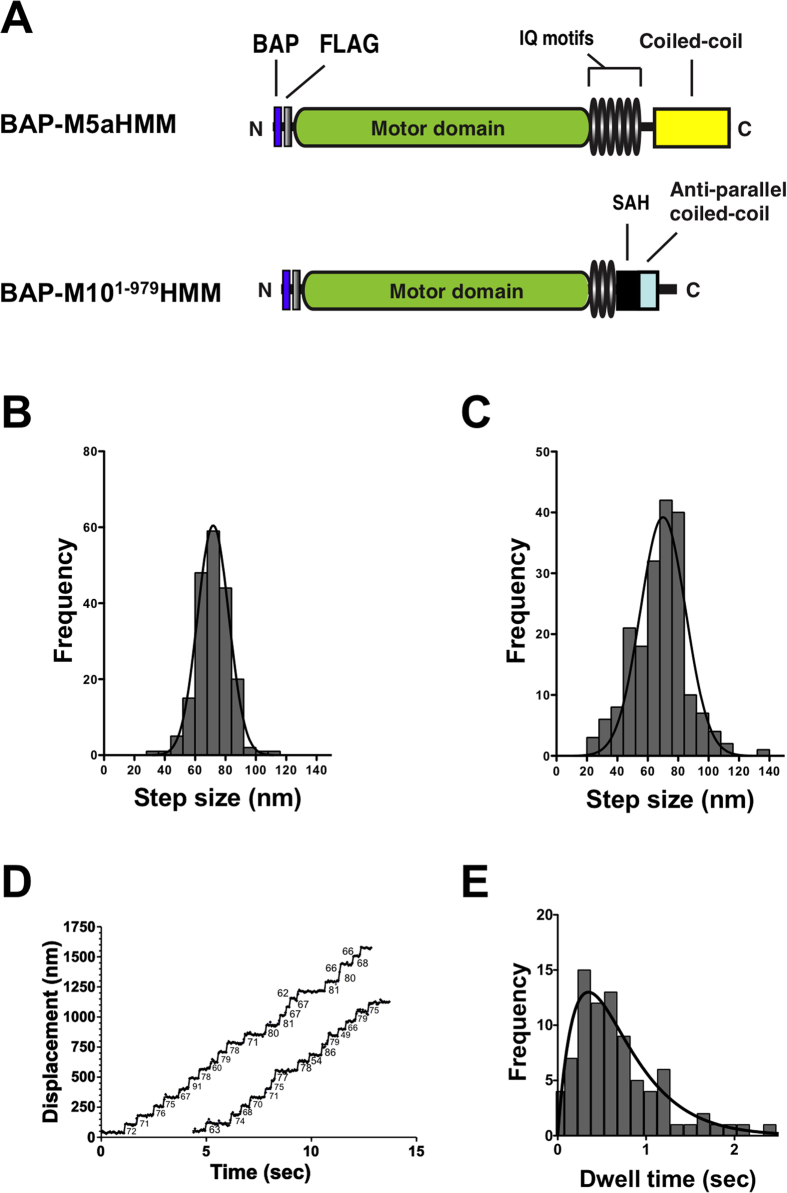
Step size distributions of N-terminal Qdot-labeled myosin Va and native myosin-X HMM constructs. (**A**) Cartoon of domain structures of BAP-M5aHMM and BAP-M10^1–979^HMM. (**B**) Step size distribution of myosin Va HMM. Experiments were done in the presence of 10 μM ATP, and the fluorescence images were captured at 16.7 fps. The mean step size of forward steps and backward steps are 71.5 ± 12.8 nm (mean ± s.d., n = 336) and −32.3 ± 12.4 nm (s.d., n = 18), respectively. The histogram was fitted with a Gaussian equation (solid line). The backward steps are not shown in the figure. (**C**) Step size distribution of BAP-M10^1–979^ HMM in the presence of 2 μM ATP. The mean step size of forward and backward steps are 67.3 ± 19.2 nm (mean ± s.d., n = 210) and −30 ± 9.6 nm (s.d., n = 4), respectively. The histogram was fitted with a Gaussian equation (solid line). The backward steps are not shown. (**D**) Representative traces of BAP-M10^1–979^HMM stepping in the presence of 2 μM ATP. Solid line represents the best fit to the trajectory. The numbers in the panel are the displacement of each step (nm). (**E**) Dwell time distribution of BAP-M10^1–979^HMM in the presence of 2 μM ATP. Solid line shows the best fit to an exponential equation, *tk*^2^e^−*kt*^, where *t* is time, and *k* is rate constant. The average waiting time (2*τ*) is 0.70 ± 0.05 s (s.e.m., n = 84).

To examine the large steps derived from myosin-X dimer derived from innate coiled-coil, we observed the step size distribution of N-terminal Q-dot-tagged myosin-X HMM without LZ (BAP-M10^1–979^HMM) (Fig. 3). Sf9 cells were co-infected with the myosin-X heavy chain-expressing virus and with the calmodulin-expressing virus as was done for M10^Full^LZ. Streptavidin-Qdot was attached to the N-terminal end of isolated M10^1–979^HMM through BAP sequence to avoid the possible disruption of dimer formation via endogenous coiled-coil domain of myosin-X. Note that this construct provides the step size of each head, therefore the obtained step size is expected to be double of the construct with Q-dot at the C-terminal end. Using a TIRF microscope, we observed the successive continuous movement of BAP-M10^1–979^ HMM on single actin filaments upon addition of 2 μM ATP to acto-BAP-M10^1–979^HMM. The result suggests that BAP-M10^1–979^HMM can form a dimer with endogenous coiled-coil domain. It was previously observed for myosin VI and myosin-X that the endogenous coiled-coil domain can form a dimer when the constructs associate with F-actin in the absence of ATP to facilitate a dimer formation^15^,^23^. The average waiting time (2*τ*) of BAP-M10^1–979^HMM at 2 μM ATP was 0.70 ± 0.05 s (s.e.m., n = 84) (Fig. 3E), which yields the calculated velocity of ~0.1 μm/s. This is consistent with the actual velocity at 2 μM ATP (0.106 ± 0.003 μm (s.e.m., n = 93)). The waiting time of BAP-M10^1–979^HMM was similar to, but slightly faster than that of M10^Full^LZ (*τ* = 0.35 s for BAP-M10^1–979^HMM vs. 0.49 s for M10^Full^LZ at 2 μM ATP). The step size distribution of BAP-M10^1–979^HMM was asymmetrical wide distribution with notably long step sizes (Fig. 3C and D) in contrast to BAP-M5HMM labeled with Qdots at the N-terminus, which showed a symmetrical step size distribution without long step size (Fig. 3B). The mean step size of BAP-M10^1–979^HMM was 67 ± 19 nm (mean ± s.d., n = 210). This result indicates that each myosin-X head takes a ~34 nm step, and the wide distribution also supports the view that the myosin-X dimer with the innate coiled-coil could take the large >50 nm step, although the most of them seem to be from the split dimer, in which the two heavy chains are connected at the C-terminal end of the molecule with LZ motif.

### Movement of M10^Full^LZ on filopodia

**Figure 4:**
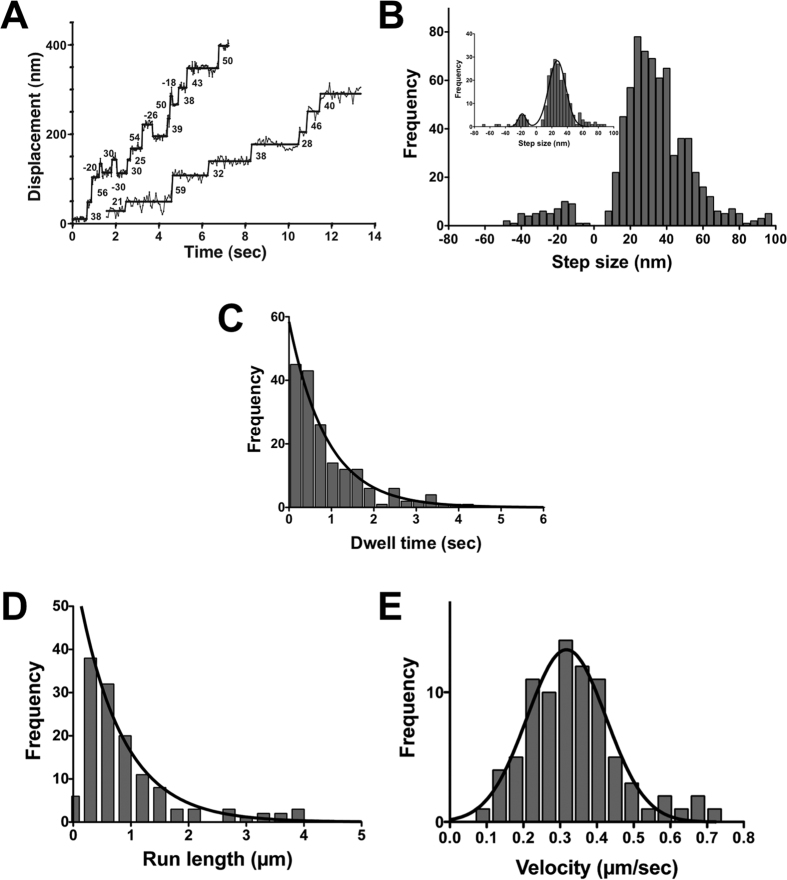
Processive movement of full-length myosin-X dimer on filopodia. (**A**) A representative trace of M10^Full^LZ stepping. The left and right traces are from 2 μM ATP and 1 μM ATP, captured at 33.3 fps and 16.7 fps, respectively. Solid line represents the best fit to the trajectory. (**B**) Step size distribution of M10^Full^LZ in the presence of 2 μM ATP. The mean step sizes of forward and backward steps are 35.8 ± 16.5 nm (s.d. n = 660) and −23.9 ± 10.8 nm (s.d., n = 56), respectively. The inset shows the step size distribution of M10^1–939^M5CC on filopodia. The mean step size of forward and backward steps are 31 ± 15 nm (mean ± s.d., n = 207) and −24 ± 14 nm (mean ± s.d., n = 24), respectively. Black solid line shows the best fit to Gaussian equation. (**C**) Dwell time distribution of M10^Full^LZ in the presence of 2 μM ATP. Solid line shows the best fit to the single exponential equation as described in Fig. 2. The average waiting time (*τ*) is 0.89 ± 0.06 s (s.e.m., n = 176). (**D**) Run length of M10^Full^LZ in the presence of 2 mM ATP. The fluorescence images were captured at 10 fps. Solid line shows the best fit to a single exponential equation, and the average run length was 0.77 ± 0.06 μm (s.e.m., n = 133). (**E**) The velocity of M10^Full^LZ in the presence of 2 mM ATP. The best fit to Gaussian equation gives the mean velocity of 0.317 ± 0.002 (s.e.m., n = 146).

**Figure 5:**
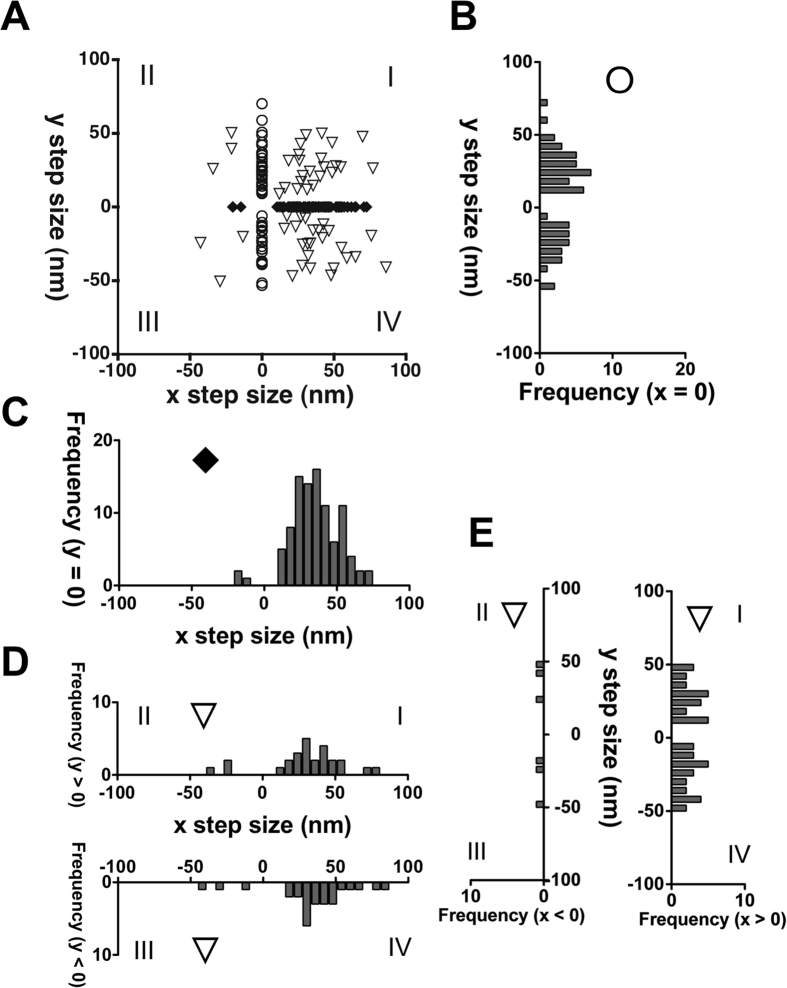
Two-dimensional stepping analysis of full-length myosin-X dimer on filopodia at 2 mM ATP. The fluorescence images of M10^Full^LZ-Qdot655 were captured at 100 fps. (**A**) Cartesian coordinate plot of M10^Full^LZ. The x and y step sizes were concurrently identified, and scattered in quadrant planes (n = 205). M10^Full^LZ can take steps along x axis (open circles, n = 96), along y axis (solid diamonds, n = 56), and diagonal steps (open reverse triangles, n = 53). Numbers of quadrant planes are denoted with Roman numerals. (**B**) Histogram of y step size at x = 0 (positive steps = 34, negative steps = 22). The mean step sizes of positive y steps and negative y steps were 26.2 ± 1.4 nm (s.e.m., ς = 12.8) and −22.7 ± 1.2 nm (s.e.m., ς = 11.9), respectively. (**C**) Histogram of x step size at y = 0 (positive steps = 93, negative steps = 3). The mean step size of forward x steps was 36.1 ± 1.5 nm (s.e.m., ς = 14.1). (**D**, upper) histogram of x-projected step size at y > 0 (positive steps = 23, negative steps = 3) The mean step size of forward x steps was 37.2 ± 3.4 nm (s.e.m., ς = 16.1). (**D**, lower) histogram of x-projected step size at y < 0 (positive steps = 24, negative steps = 3). The mean step size of forward x steps was 40.4 ± 3.6 nm (s.e.m., ς = 17.9). (**E**, left) histogram of y-projected step size at x < 0 (positive steps = 3, negative steps = 3); (**E**, right) histogram of y-projected step size at x > 0 (positive steps = 23, negative steps = 24). The mean step sizes of positive y-projected steps and negative y-projected steps were 27.8 ± 2.6 nm (s.e.m., ς = 12.4) and −25.4 ± 3.2 nm (s.e.m., ς = 13.1), respectively. The statistical calculation of the distribution with small data number is not shown.

As mentioned above, myosin-X travels along filopodia in cells^4^,^5^. To study the movement of full-length myosin-X in physiological set-up, we examined the movement of M10^Full^LZ in filopodia of demembranated MEF3T3 cells. MEF3T3 cells were transfected with cdc42 expression vector to induce filopodia (Supplementary Fig. 4A). The cells were then treated with Triton-X100 to remove surface membrane, and stained with Alexa phalloidin as described in Methods. M10^Full^LZ -Qdot was then applied on the demembranated cells. After being washed with the blocking buffer in PBS, the motility buffer containing 2 μM ATP or 2 mM ATP was applied to the cells (Supplementary Fig. 4B). M10^Full^LZ-Qdot primarily moved on filopodia and we could not find them moving on other cytoskeletal structures (Supplementary Fig. 4B and Supplementary Movie 1). The FIONA analysis at 2 μM ATP revealed the successive multiple stepping of M10^Full^LZ in filopodia (Fig. 4A). The mean step size of forward and backward steps of x-projection were 35.8 ± 16.5 nm (mean ± s.d., N_steps/Qdots/filopodia/cells_ = 660/90/70/35) and −23.9 ± 10.8 nm (s.d., N_steps/Qdots/filopodia/cells_ = 56/33/28/21), respectively (Fig. 4B). The mean forward step size was smaller than those found in *in vitro* single actin filaments (Fig. 2B), which is likely to be due to the presence of the y-component of the stepping during the movement in filopodia (see Fig. 5). The step size distribution was asymmetrical and broad unlike the tail-truncated forced dimer construct of myosin-X (Fig. 4B inset), and was best explained with two different step sizes, i.e., ~27 nm and ~50 nm. Dwell time distribution in filopodia showed the average waiting time (*τ*) of 0.89 ± 0.06 s (s.e.m.), which is twice longer than that on single actin filaments at 2 μM ATP (Fig. 4C). This indicates that it takes a longer time to find the landing point on filopodia than on single actin filaments. The movement of M10^Full^LZ on filopodia was also tested at 2 mM ATP (Fig. 4D). The average run length was 0.77 ± 0.06 μm (s.e.m., 0.691 ± 0.006 μm from survival curve, see also Supplementary Fig. 5Bb), which is similar to that at 2 mM ATP on single actin filaments (Fig. 2E). The mean velocity of M10^Full^LZ on filopodia is 0.317 ± 0.002 μm/s (s.e.m., Fig. 4E), similar to the velocity on single actin filaments (Fig. 2F).

**Figure 6:**
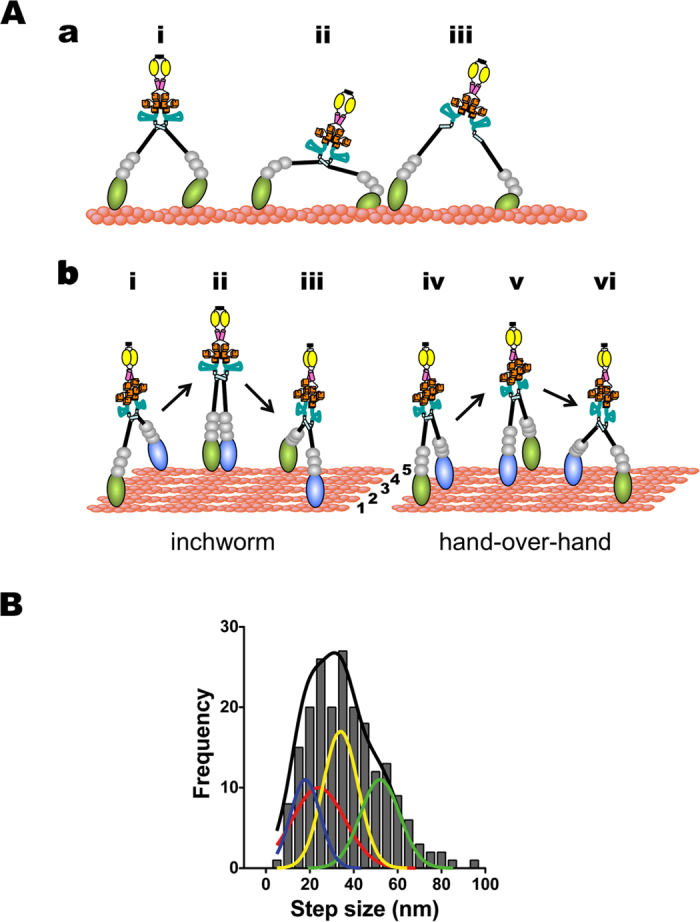
Model of stepping pattern of myosin-X on filopodia. (**A**) Full-length myosin-X steps. (a) The x forward steps of M10^Full^LZ on filopodia. Half pitch = 36 nm (i) and 3/4 pitch = 54 nm (ii and iii) are shown. Note that a part of coiled-coil of myosin-X molecules is possibly open as shown in (iii), and that the landing position at 54 nm can be a side of the actin filament. (b) The y-steps of M10^Full^LZ on filopodia. It is assumed that full-length myosin-X takes y-steps (i and iv) and diagonal steps (iii and vi) with the inchworm-like mechanism (i, ii and iii) and hand-over-hand mechanism (iv, v and vi). For the inchworm model, myosin-X takes the 24 nm y-step across the 5 actin filaments (i and ii), while the 24 nm step size corresponds to the space between 3 actin filaments in the hand-over-hand mechanism (iv and v). The 36 nm x-steppings and 24 nm y-steppings are assumed to occur simultaneously for the diagonal steps (iii and vi). The numbers of 1–5 actin filaments are shown. (**B**) Histogram of M10^Full^LZ step size in all directions. The distance of all x-y position from the origin in Fig. 5 was calculated and made the histogram. Black solid line is the sum of four Gaussian equations, when assumed that myosin-X takes 18 nm, 24 nm, 36 nm, and 54 nm step sizes. The area ratios of independent Gaussian distribution are ~17% for 18 nm (blue), ~28% for 24 nm (red), ~32% for 36 nm (yellow) and ~23% for 54 nm (green).

Since filopodia is composed of F-actin filament bundles, we studied two-dimensional movement of M10^Full^LZ at 2 mM ATP (Supplementary Fig. 6). Figure 5 shows a Cartesian plot analysis of M10^Full^LZ movements (N_steps/Qdots/filopodia/cells_ = 205/13/12/9). While M10^Full^LZ primarily steps towards the tip of filopodia (x-step size), the displacement to vertical or diagonal direction is also observed (Fig. 5A). For y-steps, 34 positive steps and 22 negative steps were observed (Fig. 5B). The mean step sizes of forward x-steps were 36.1 ± 1.5 nm (s.e.m., Fig. 5C). This value is similar to a half-pitch of F-actin helix (~36 nm) and ~34 nm step size of BAP-M10^1–979^HMM on single actin filaments (Fig. 3B), although large step sizes (>50 nm) were observed. On the other hand, the mean step sizes of positive y-steps and negative y-steps were 26.2 ± 1.4 nm (s.e.m.), and −22.7 ± 1.2 nm (s.e.m.). These values coincide with the space between the two actin filaments ~24 nm apart (2 × 11–13 nm space between the adjacent filaments in fascin bundles^24^) from each actin (see Fig. 6Ab) in fascin-based actin bundles. The presence of y-steps in part explains the decrease in the overall mean step size of M10^Full^LZ on filopodia. We also found a significant number of diagonal displacements (Fig. 5D and E). The values in Fig. 5D and E are the x- and y- projection of the diagonal steps, respectively. These results suggest that M10^Full^LZ moves various direction in filopodia, which is consistent with the flexible nature of the myosin-X motor^15^. It should be noted that M10^Full^LZ continuously moved after y-stepping or diagonal stepping towards filopodial tips. Sometimes we observed successive multiple y-stepping and diagonal stepping prior to x-stepping towards filopodial tips.

### Visualization of M10^Full^LZ molecules by electron microscopy

Electron microscopy was employed to clarify the molecular behaviors of the M10^Full^LZ molecules in the presence of the actin. Supplementary Figure 7A shows the general appearances of M10^Full^LZ molecules in single molecular state. According to appeared inter-head distances of two-headed molecules, there are two main conformations of M10^Full^LZ molecules visualized in the fields: shorter and longer lengths of the inter-head conformers, (inset image in Supplementary Fig. 7A). Similar observations of the inter-head distances of the single molecules were found in the presence of the actin (Supplementary Fig. 7B) and the inter-head lengths from the two main appearances of the molecules binding to actin filament were measured in the images (inset image in Supplementary Fig. 7B). Measured mean ± s.d. lengths of shorter and longer inter-head distances in the molecules are 35.7 ± 0.8 nm (21 particles) and 53.9 ± 1.4 nm (10 particles). Taken together with the step length measurements as shown in Fig. 5, this result demonstrates that M10^Full^LZ molecules can form the shorter and longer lengths of the inter-head conformers when interacted with the actin filaments.

## Discussion

Our aim of this study is to clarify the authentic nature of the movement of myosin-X on actin filaments. To address this question, we generated a full-length myosin-X construct and analyzed its movement at a single molecule level on both single actin filaments and filopodial actin bundles in cells. Since a full-length myosin-X is monomeric and inhibited^19^, we studied the motility in the presence of PI(3,4,5)P_3_, which activates the full-length myosin-X^19^. As we reported previously, a full-length myosin-X processively moves within filopodia in living cells at singe molecule level^6^,^25^,^26^. The result suggests that a full-length myosin-X forms a dimer in cells. However, the concentration of overexpressed myosin-X in living cells is much higher than that of *in vitro* single molecule experiment, in which pM concentration of myosin-X is used. In order to facilitate inter-heavy chain interaction at the coiled-coil domain of myosin-X, we introduced a LZ at the C-terminal end of the tail. The produced full-length myosin-X showed processive movement on actin filaments, however, unlike myosin Va, the step size distribution is broad and asymmetric for both single actin filaments and filopodia. Our result suggests that this is because there are multiple step sizes of myosin-X moving on actin filaments. The step size distribution was best explained with multiple step sizes. Electron microscopy revealed that M10^Full^LZ exhibits two configurations of different inter-head distance (Supplementary Fig. 7), which correspond to the multiple step sizes found in the present study.

An important novel point in this study is the observation of two-dimensional movement of single myosin-X molecules on filopodia (Fig. 5). The step size distribution along x-axis (mean = ~36 nm) was quite similar to the distributions on single actin filaments (Fig. 2) and on filopodia (Fig. 4). The distribution of y-step was broad, and the mean y-step was close to ~24 nm. If myosin-X steps along the y-axis at ~24 nm, the myosin-X leading head at the first step will land about 5 actin filaments apart from the trailing head (Fig. 6Ab). The subsequent ~24 nm y-step can be achieved by the inchworm stepping of the trailing head. Alternatively, ~24 nm y-steps can be due to the hand-over-hand stepping on every other actin filaments in the bundles. From the analysis of diagonal steps, we found that the y-step components were relatively constant (~24 nm), irrespective of the x-step sizes (Supplementary Table 1). Taken these findings together, the results suggest that the myosin-X leading head prefers to step on the fifth/or third actin filaments apart from the trailing head in filopodia.

Of interest is the production of >50 nm x-steps. The wide stepping distribution is also observed in artificially elongated myosin Va HMM^27^. The result suggests that the landing position of the myosin head can be the side of actin filaments. This suggests that the lever-arm of full-length myosin-X is flexible enough to step on various positions on actin filaments. We asked whether the >50 nm steps occur during the course of multiple stepping or it only occurs at the final step. The most >50 nm steps are observed between normal steps. As shown in Fig. 2A, the successive multiple >50 nm steps were also observed. The results suggest that the >50 nm steps are mechanically active steps. It is reported that the myosin-X lever arm with 3 IQ motifs is extending about 10.8 nm on F-actin filament^15^. Assuming that the postulated SAH domain of myosin-X is ~15 nm^28^, the entire lever arm of 2 myosin-X heads is expanded by about 51.6 nm when myosin-X is dimerized. When myosin-X forms an antiparallel dimer, an additional 4.7 nm is incorporated to the span between the two heads. Therefore, it is plausible that >50 nm steps are arisen from the myosin-X antiparallel dimer (Fig. 6Aa).

Several studies of single molecule analysis of a tail-truncated forced dimer of myosin-X have been reported, and the step size of myosin-X is controversial^14^,^15^,^17^,^18^,^22^. We previously reported^15^ that myosin-X step size is 34 nm on single actin filaments and 18 nm and 34 nm on fascin-actin bundles, while Nagy *et al*.^22^ and Ricca and Rock^17^ reported ~18 nm step size of myosin-X for both single actin filaments and fascin-actin bundles, but not 34 nm step size, and claimed that the difference is due to in or out of register with the heptad repeat of intrinsic coiled-coil and the connecting artificial coiled-coil. Their construct has 1–920 amino acids of native myosin-X with a leucine zipper motif right after the myosin-X’s coiled-coil with in-register while our HMM construct has 1–939 amino acids of native myosin-X, followed by myosin V’s coiled-coil. While the high score of coiled-coil probability ends around 920, we think that the forced dimer motif should be far from the intrinsic coiled-coil to avoid possible disruption of the intrinsic coiled-coil conformation of myosin-X. Lu *et al*.^9^ previously reported that myosin-X’s coiled-coil makes an anti-parallel dimer, and may alter the dimer structure when a forced dimer is introduced right after myosin-X’s intrinsic coiled-coil. Takagi *et al*.^18^ recently reported that the single molecule myosin-X 1–936 HMM followed by leucine zipper showed a ~17 nm lever arm displacement, which cause a ~35 nm forward step size in an optical tweezers assay. This study agrees well with our previous study^15^, and suggests that at least 16 amino acids sequence after 920th of myosin-X may affect the step size of myosin-X. One of the best approaches to circumvent this problem and address the innate motor property of myosin-X is the use of full-length myosin-X. The present study shows that a full-length myosin-X moves predominantly with 38 nm step size (Fig. 2B). Another approach is the use of myosin-X with only the innate myosin-X coiled-coil. Our study also shows that myosin-X HMM without an artificial dimer structure moves with ~34 nm, which is a half size of the 67 nm observed in single head-labeled BAP-M10^1–979^HMM (Fig. 3C). These results indicate that the authentic step size of myosin-X is predominantly 34–38 nm.

It should be noted while our results do not show the apparent peak of ~18 nm step size in the step size distribution analysis, the data can be explained with the presence of 18 nm step size in addition to 36 nm step size and 54 nm step size (Fig. 6B). The mechanism for the ~18 nm step size of myosin-X is obscure, but hand-over-hand stepping is less likely on single actin filaments considering the long length of the lever-arm. It is rather plausible that ~18 nm steps are arisen from inchworm-like stepping, as seen in full-length myosin Va in EGTA condition^29^.

M10^Full^LZ increased the number of filopodia (Supplementary Fig. 2), and the filopodia length is much longer than that induced by the tail-truncated myosin-X forced dimer (data not shown). Furthermore, the effects of M10^Full^LZ on filopodia formation and elongation are similar to those of wild-type full-length myosin-X (data not shown), suggesting that M10^Full^LZ retains the authentic properties of myosin-X. The full-length myosin-X shows a ~38 nm mean step size on single actin filaments, and ~36 nm on filopodia. A slight decrease in the mean step size in filopodia is in part due to the presence of y steps (vertical to actin filaments). This is consistent with our previous report^15^, where *in vitro* fascin-actin bundles were used for the step size analysis. In order to study the movement of myosin-X in near physiological environment, we examined the myosin-X movement in filopodia using a demembranated cell system^30^,^31^. Our results show that the full-length myosin-X produces a significant number of y-directional stepping in addition to x-directional (towards filopodial tips) stepping in filopodia. These steppings are followed by x-directional, y-directional or diagonal stepping, indicating that these steppings represent actual movement of myosin-X. This finding suggests that myosin-X is a motor that can step to various angles in filopodia, which enables myosin-X to bridge different actin filaments in cells. This property is likely to be in part due to the flexibility of the lever-arm of myosin-X^15^, and it is plausible that this nature enables myosin-X to converge actin filaments to initiate filopodia. Schematic drawing of the possible conformations of M10^Full^LZ is shown in Fig. 6A, and the over-all step size distribution of M10^Full^LZ including y-stepping on filopodia is shown in Fig. 6B. The mean step size of myosin-X in all directions was calculated to be 36.6 ± 16.7 nm (s.d., n = 205). The step size distribution is widely distributed, which is likely to be due to the presence of multiple step sizes including 34–36 nm stepping^14^,^15^,^18^,^32^, 24 nm y-stepping^This study^, >50 nm large stepping^This study^ and possibly ~18 nm stepping^15^,^17^,^22^. We think that the large >50 nm step size can be in part arisen from an antiparallel dimer of myosin-X with innate coiled-coil. The result of EM observation (Supplementary Fig. 7) is consistent with this view. However, while the step size observation of BAP-M10^1–979^HMM without LZ shows wide step size distribution and notably more fractions of >90 nm size than that of BAP-M5HMM (Fig. 3B), the fraction of large step sizes is less than that of M10^Full^LZ. This may indicate that an open conformation of the molecule (see Fig. 6Aa) predominantly contributes to the >50 nm large steps.

During the preparation of this paper, Ropers *et al*.^32^ reported that full-length myosin-X moves on single actin filaments with ~36 nm step size, and on synthetic fascin-actin bundles with 19 nm, 38 nm and 52 nm steps. Consistent with the present work, full-length myosin-X showed the broad step size distribution on single actin filaments. However, the step size distribution on fascin-actin bundles is somewhat different from our data using filopodial actin. Moreover, the velocity of their full-length myosin-X is twice faster and the run-length is 2.4-fold longer in fascin-actin bundles, compared with those on single actin filaments. In this study, we could not find any substantial differences between the movements on single actin filaments and on filopodia. Although the reason of apparent difference remains to be elucidated, it is plausible that these differences are due to the difference of actin structures between the reconstituted fascin-actin bundles and filopodia.

By using synthetic fascin-actin bundles, it was reported that the run length of a tail-truncated forced dimer of myosin-X HMM on fascin-actin bundles is much longer than that on single actin filaments^14^,^22^,^32^. In this study, however, the mean run length of full-length myosin-X in filopodia (0.77 μm at 2 mM ATP, Fig. 4D) is similar to that on single actin filaments (0.78 μm at 2 mM ATP, Fig. 2E). This is consistent with the results of Sun *et al*.^15^, who reported that run length of myosin-X HMM is 0.95 μm on single actin filaments and 1.16 μm on fascin-actin bundles, respectively^15^. The apparent difference in the results can be in part due to the difference in the actin structure and it is plausible that the fascin-induced synthetic actin bundles are different from filopodial actin bundles presumably due to the difference in the structure with additional proteins in filopodial actin structure. Further studies are required to clarify the effect of additional filopodial components on intra-filopodial myosin movement.

## Materials and Methods

Rhodamine-phalloidin and streptavidin conjugated Qdots were purchased from Invitrogen. Actin was purified from rabbit skeletal muscle according to Spudich *et al*.^33^.

Bovine myosin-X cDNA fragments were kindly provided by Dr. D. P. Corey (Harvard University). Baculovirus expressing recombinant full-length bovine myosin-X (M10^Full^) (including 1–2052, amino acids) was made as previously described^19^. The stop codon of M10^Full^ was removed and converted to Mlu 1 site by PCR site-directed mutagenesis^34^ using Pfu Ultra II (Agilent Technologies), and an oligo DNA containing c-Myc sequence (amino acid: EQKLISEEDL) was inserted between Mlu1 sites. A GCN4 leucine zipper sequence^35^ after a linker motif sequence (amino acid: GGGSGGGSGGGS) was then inserted before the c-Myc tag motif. The full-length myosin-X with leucine zipper (M10^Full^LZ) was subcloned into a modified pFastBacHT baculovirus transfer vector (Life technologies) having a FLAG sequence between His tag and Tev protease site. For experiments of myosin-X HMM, we constructed myosin-X HMM (1–939 residues) with myosin Va coiled-coil^5^,^15^, and inserted biotin acceptor peptide (BAP) sequence (GGGLNDIFEAQKIEWHE) at the C-terminal ends. Myosin-X native HMM construct (BAP-M10^1–979^HMM) was also made by inserting BAP sequence instead of GFP at N-terminal side of myosin-X HMM^15^. For myosin Va experiments, we produced mouse myosin Va HMM (amino acids, 1–1105) with BAP sequence at the C- or N-terminal sides. M10 proteins were expressed in Sf9 cells by co-infection of myosin-X heavy chain with CaM viruses, and prepared using anti-FLAG antibodies affinity column chromatography as described^36^.

### Labeling of myosin-X and F-actin

For M10^Full^LZ experiments, either Qdot 525 (Q11022MP, Invitrogen) or Qdot 655 (Q11041MP) conjugated with goat F(ab’)_2_ anti-mouse IgG (H + L) were mixed with anti-c-Myc monoclonal antibodies (Clontech) at the Qdot/antibody ratio of 0.85: 1, and incubated at 4 °C for >1 day. M10^Full^LZ was then labeled with the Qdot–antibody by mixing and incubating them on ice for >1 hour before experiments. Biotinylated M10^1–939^M5CC was prepared and mixed with streptavidin–conjugated Qdot 525 (Q110141MP, Invitrogen) or Qdot 655 (Q10121MP) as described previously^15^. To ensure single molecule conditions, the myosin-X/Qdot ratio was usually adjusted to 1: 20 unless otherwise indicated. Under this condition, it is calculated from Poisson statistics that ~9% and ~90% of the myosin-X would be occupied with one and two Qdots at the C-terminus, respectively. We didn’t distinguish one or two Qdots/myosin-X in the present study. Instead, we didn’t adopt the data from extra bright fluorophores, which is probably composed of 3 or more Qdots from multi-heads of myosin-X. For M10^Full^LZ-Qdot 655 experiments, we adopted myosin-X/Qdot ratio of 1:5 by comparing first-power landing rates between myosin-X-Qdot 525 and myosin-X-Qdot 655 to maintain single molecule conditions as previously reported^17^. The proximity-induced dimerization of BAP-M10^1–979^HMM (myosin-X HMM with native coiled-coil) was carried out according to Sun *et al*.^15^. The dimerized BAP-M10^1–979^HMM was labeled with streptavidin-conjugated Qdot 655 with 1:4 ratio. F-actin was sparsely labeled with rhodamine-phalloidin as previously described^36^. For myosin Va experiments, myosin Va HMM biotinylated at C-terminus and streptavidin–conjugated Qdot 655 were mixed at the ratio of 1:20 before observations. The myosin Va HMM biotinylated at N-terminus was mixed with streptavidin-conjugated Qdot 655 at 1:4 ratio as described^37^.

### Cell preparation

For preparation of extracted cells, MEF3T3 cells were treated with extraction buffer as described previously^31^ with slight modifications. In brief, the cells were treated with extraction buffer (30 mM imidazole, pH 7.5, 70 mM KCl, 1 mM EGTA, 2 mM MgCl_2_, 0.5% Triton X-100, 4% polyethylene glycol (mol wt 8,000), and 250 nM Alexa Fluor 568 phalloidin (invitrogen)) for 4 min on ice and then washed twice with ice cold PBS. All of the samples were incubated on ice with blocking solution (1% BSA, 0.2 mg/ml streptavidin and 1 mg/ml casein in PBS) before use in single molecule assays.

### Single molecule TIRF motility assay

The movement of myosin-X was observed with a home-built TIRF setup using an inverted Olympus IX71 microscope, an objective lenz (PlanApo-N × 60, 1.45 numerical aperture, oil), 473 nm diode–pumped solid state laser (model BL-9040, PHOTOP) or 488 nm from an Argon/Krypton ion laser (model 35 LDL 840-208, Melles-Griot), and a back-illuminated electron multiplier CCD camera (model iXON DU-897E or DU-860E, Andor Technology). For the experiments of single actin filaments, flow chambers were prepared by using No. 1.5 glass coverslips and glass slides (Fisher Scientific). Alpha-actinin (A9776, SIGMA) was used to immobilize F-actin, and casein from bovine milk (07319-82, Nakarai Tesque, Japan) and/or streptavidin (Thermo Scientific) was used for glass-surface blocking. The movement of myosin-X was observed in a solution containing 25 mM KCl, 20 mM HEPES (pH 7.5), 5 mM MgCl_2_, 1 mM EGTA, 5 mM DTT, 12 μM calmodulin, and an O_2_ scavenging system containing glucose oxydase, catalase and glucose in the presence of indicated concentration of ATP. For M10^Full^LZ experiments, 5 μM PI(3,4,5)P_3_ diC8 (P-3908, Echeron) was added to the assay solution to activate myosin-X motor activity. The PI(3,4,5)P_3_ diC8 does not make micelles at 5 μM (CMC >3 mM). All the experiments were done at 24 °C.

### DATA analysis

The captured images were analyzed by using in-house 2D Gaussian fitting software^6^. If necessary, a function for the recorded stage drift was calculated and subtracted from the raw data of each runs for each movie. Run length and velocity of myosin-X were concurrently determined by measuring the length and time between on and off the tracks. Step size and dwell time were determined by using a step-fitting software based on an algorithm described in Kerssemakers *et al*.^38^. The average number of frames in each step was 16.3 for M10^Full^LZ (2 μM ATP) on single actin filaments, and the typical position of stationary myosin-X-Qdot on actin filaments was 0.0 ± 2.5 nm (mean ± s.e.m., ς = 10.2), indicating that the tracking precision was <3 nm under the typical experimental condition. For the step size analysis in the presence of 2 mM ATP, we chose myosin-X molecules with a slow speed (0.1–0.2 μm/s). For the Cartesian coordinate plot, the x step size and y step size were independently determined and plotted to the quadrant planes as described previously^17^.

### *In vitro* multi-molecule assay

*In vitro* motility assay was carried out according to the method previously described using IX71 microscope with a motorized filter wheel (ProScan II, Prior Scientific) and a CCD camera (Sensicam, PCO/Cooke)^19^,^36^.

### Electron Microscopy

For negative staining, M10^Full^LZ (300 nM) was mixed with 6 μM of calmodulin (ratio of 1 to 20 to enhance calmodulin binding) and then 10-fold diluted with low salt buffer containing 50 mM Na acetate, 1 mM EGTA (1.1 mM CaCl_2_), 2 mM MgCl_2_, 10 mM MOPS, 200 μM ATP, and pH7.5. To visualize M10^Full^LZ molecules attached to F-actin, specimens were made by mixing 300 nM of M10^Full^LZ molecules and 6 μM of calmodulin with an equal volume of 10 μM of F-actin, then 10-fold diluted with above low salt buffer. After dilution, 5 μl of the final mixture were applied to a carbon-coated grid that had been glow-discharged (Harrick Plasma, Ithaca, NY) for 3 min in air, and the grid was immediately (~5 sec) negatively stained using 1% uranyl acetate^39^. Grids were examined in a Technai 10 TEM (FEI, USA) operated at 100 keV and images were recorded at a magnification of 34,000 (0.32 nm per pixel).

### Confocal light microscopy

Localization of myosin-X in COS7 cells was observed by using a laser scanning confocal microscope, SP2 system (Leica Microsystems, Heidelberg, Germany).

## Additional Information

**How to cite this article**: Sato, O. *et al*. Activated full-length myosin-X moves processively on filopodia with large steps toward diverse two-dimensional directions. *Sci. Rep.* **7**, 44237; doi: 10.1038/srep44237 (2017).

**Publisher's note:** Springer Nature remains neutral with regard to jurisdictional claims in published maps and institutional affiliations.

## Supplementary Material

Supplementary Information

Supplementary Movie 1
