# Supplementary material for: Activated full-length myosin-X moves processively on filopodia with large steps toward diverse two-dimensional directions
Source: Sci Rep. 2017 Mar 13;7:44237. doi: 10.1038/srep44237 (PMC5346999; doi:10.1038/srep44237)
Supplement: Supplementary Information [file srep44237-s1.pdf]

Activated full-Length Myosin X moves processively on filopodia with large steps toward diverse two-dimensional directions.

Osamu Sato[1^], Hyun Suk Jung[2^], Satoshi Komatsu[1], Yoshikazu Tsukasaki[1,3], Tomonobu M. Watanabe[1,4], Kazuaki Homma[1,5], and Mitsuo Ikebe[1*]

[1]Department of Cellular and Molecular Biology, University of Texas Health Science Center at Tyler, Tyler, Texas 75708, USA
[2]Department of Biochemistry, College of Natural Sciences, Kangwon National University, Chuncheon-si, Gangwon-do 24341, Korea
[3]Department of Pharmacology, University of Illinois College of Medicine, Chicago, Illinois 60612, USA
[4]Laboratory for Comprehensive Bioimaging, RIKEN Quantitative Biology Center, Suita, Osaka 565-0874, Japan
[5]Department of Otolaryngology-Head and Neck Surgery, Feinberg School of Medicine, Knowles Hearing Center, Northwestern University, Chicago, Illinois 60611, USA

^ These authors equally contributed to this work.
*Correspondence should be addressed to this author.

Supplementary figures and Tables

1. **Supplementary Figure 1**

2. **Supplementary Figure 2**

3. **Supplementary Figure 3**

4. **Supplementary Figure 4**

5. **Supplementary Figure 5**

6. **Supplementary Figure 6**

7. **Supplementary Figure 7**

8. **Supplementary Table 1**

9. **Legend to Supplementary Movie 1**

A

M10^{Full}LZ

```
1     MSYYHHHHHHDYKDDDDKNIPTTENLYFQGAMGIRNSKAYVDMDNFFPEGTRVWLRENGQHFPSTVNSCAEGVVVFQTDY
          His-FLAG-Tev sites                          Motor domain
81    GQVFTYKQSTITHQKVMPMQPTDEEGVDDMATLTELHGGAIMHNLYQRYKRNQIYTYIGSIIASVNPYKTITGLYSRDAV
161   DRYSRCHLGELPPHVFAIANECYRCLWKRHDNQCVLISGESGAGKTESTKLILKFLSAISQQSVDLSSKEKTSSVEQAIL
241   ESSPIMEAFGNAKTVYNNNSSRFGKFVQLNIGQKGNIQGGRIVDYLLEKNRVVRQNPGERNYHIFYALLAGLGHEEREEF
321   YLSVPENYHYLNQSGCVTDRTISDQESFREVIMAMEVMQFSKEEVREVLRLLAGILHLGNIEFITAGGAQVSFKTALGRS
401   AELLGLDPAQLTDALTQRSMFLRGEEILTPLNVQQAADSRDSLAMALYARCFEWVIKKINSRIKGKDDFKSIGILDIFGF
481   ENFEVNHFEQFNINYANEKLQEYFNKHIFSLEQLEYSREGLVWEDIDWIDNGECLDLIEKKLGLLALINEESHFPQATDS
561   TLLEKLHNQHANNHFYVKPRVAVNNFGVKHYAGEVQYDVRGILEKNRDTFRDDLLNLLRESRFDFIYDLFEHVSSRNNQD
641   TLKCGSKHRRPTVSSQFKDSLHSLMATLSASNPFFVRCIKPNMQKMPDQFDQAVVVNQLRYSGMLETVRIRKAGYAVRRP
721   FQDFYKRYKVLMRNVALPEDIRGKCTALLQLYDASNSEWQLGKTKVFLRESLEQKLEKRQEEEVTRAAMVIRAHVLGYLA
                                                                              IQ motif 1
801   RKQYKKVLDCVVIIQKNYRAFLLRRRFLHLKKAAVVFQKQLRGQIARRVYRQLLAEKRAEEEKRKREEEEKRKREEEERE
                    IQ motif 2                    IQ motif 3          SAH
881   RERERREAELRAQQEEAARKQRELEALQQESQRAAELSRELEKQKENKQVEEILRLEKEIEDLQRMKERQELSLTEASLQ
                                                       Antiparallel coiled-coil
961   KLQQLRDEELRRLEDEACRAAQEFLESLNFDEIDECVRNIERSLSVGSGCTGEQGAGAEKPSFNFSQPYPEEEEVDEGFE
                                                                        PEST domain
1041  ADDDAFKDSPNPSEHGHSDQRTSGIRTSDESSEEDPYMNDTVVPTSPSADSTVLLAPSEHDSSAGEPTYCLPQTPGALPA
1121  PEGDYDYDQDDYEDGAITSGSSVTFSNSCSSQWSPDYRCSVGTYNSSGAYRFSSEGAQSSFEDSEEDFDSRFDTDDELSY
1201  RRDSVYSCVTLPYFHSFLYMKGGLMNSWKRRWCVLKDETFLWFRSKQEALKQGWLHKKGGGSSTLSRRNWKKRWFVLRQA
           PH domain
1281  KLMYFENDSEEKLKGTVEVRAAKEIIDNTSKENGIDIIMADRTFHLIAESPEDASQWFSVLSQVHASTDQEIREMHDEQA
1361  NPQNAVGTLDVGLIDSVCASDSPDRPNSFVIITANRVLHCNADTPEEMHHWITLLQRSKGDTRVEGQEFIVRGWLHKEVK
1441  NSPKMSSLKLKKRWFVLTHNSLDYYKSSEKNALKLGTLVLNSLCSVVPPDEKIFKETGYWNVTVYGRKHCYRLYTKLLNE
1521  ATRWSSAIQNVTDTKAPIDTPTQQLIQDIKENCLNSDVVEQIYKRNPILRHTHHPLHSPLLPLPYGDINLNLLKDKGYTT
                                                                         MyTH4 domain
1601  LQDEAIKIFNSLQQLESMSDPIPIIQGILQTGHDLRPLRDELYCQLIKQTNKVPHPGSVGNLCSWQILTCLSCTFLPSRG
1681  ILKYLKFHLRRIREQFPGTEMEKYALFIYESLKKTKCREFVPSRDEIEALIHRQEMTSTVHCHGGGSCKITVNSHTTAGE
1761  VVEKLIRGLAMEDSRNMFALFEYNGHVDKAIESRTIVADVLAKFEKLAATSEVGEQPWKFYFKLYCFLDTDNVPKDSVEF
                                                    FERM domain
1841  AFMFEQAHEAVIHGHYPAPEENLQVLAALRLQYLQGDYAPHAPVPPLEEVYSLQRLKARISQSTKSFTPGERLEKRRTSF
1921  LEGTLRRSFRTGSAIRQKAEEEQMVDMWVKEEVCSARASILDKWKKFQGMSQEQAMAKYMALIKEWPGYGSTLFDVECKE
2001  GGFPQDLWLGVSADAVSVYKRGEGRPLEVFQYEHILSFGAPLANTYKIVVDERELLFETSEVVDVAKLMKAYISMIVKKR
2081  YSTSRSVSSQGSSRTRPRGGGSGGGSGGGSSMKQLEDKVEELLSKNYHLENEVARLKKLVGERTREQKLISEEDL
                       Linker          GCN4 leucine zipper                   c-Myc
```

B

Bap-M10$^{1–979}$ HMM

```
1     MAGGLNDIFEAQKIEWHERSETMSYYHHHHHHDYKDDDDKNIPTTENLYFQGAMGIRNSKAYVDMDNFFPEGTRVWLREN
         BAP sequence          His-FLAG-Tev sites                        Motor domain
81    GQHFPSTVNSCAEGVVVFQTDYGQVFTYKQSTITHQKVMPMQPTDEEGVDDMATLTELHGGAIMHNLYQRYKRNQIYTYI
161   GSIIASVNPYKTITGLYSRDAVDRYSRCHLGELPPHVFAIANECYRCLWKRHDNQCVLISGESGAGKTESTKLILKFLSA
241   ISQQSVDLSSKEKTSSVEQAILESSPIMEAFGNAKTVYNNNSSRFGKFVQLNIGQKGNIQGGRIVDYLLEKNRVVRQNPG
321   ERNYHIFYALLAGLGHEEREEFYLSVPENYHYLNQSGCVTDRTISDQESFREVIMAMEVMQFSKEEVREVLRLLAGILHL
401   GNIEFITAGGAQVSFKTALGRSAELLGLDPAQLTDALTQRSMFLRGEEILTPLNVQQAADSRDSLAMALYARCFEWVIKK
481   INSRIKGKDDFKSIGILDIFGFENFEVNHFEQFNINYANEKLQEYFNKHIFSLEQLEYSREGLVWEDIDWIDNGECLDLI
561   EKKLGLLALINEESHFPQATDSTLLEKLHNQHANNHFYVKPRVAVNNFGVKHYAGEVQYDVRGILEKNRDTFRDDLLNLL
641   RESRFDFIYDLFEHVSSRNNQDTLKCGSKHRRPTVSSQFKDSLHSLMATLSASNPFFVRCIKPNMQKMPDQFDQAVVVNQ
721   LRYSGMLETVRIRKAGYAVRRPFQDFYKRYKVLMRNVALPEDIRGKCTALLQLYDASNSEWQLGKTKVFLRESLEQKLEK
801   RQEEEVTRAAMVIRAHVLGYLARKQYKKVLDCVVIIQKNYRAFLLRRRFLHLKKAAVVFQKQLRGQIARRVYRQLLAEKR
                IQ motif 1              IQ motif 2              IQ motif 3          SAH
881   AEEEKRKREEEEKRKREEEEREREREREEAELRAQQEEAARKQRELEALQQESQRAAELSRELEKQKENKQVEEILRLEK
                                                                  Antiparallel coiled-coil
961   EIEDLQRMKERQELSLTEASLQKLQQLRDEELRRLEDEACRAAQEFLESLNFDEIDECVRNIERSLSVGSGCTGEQGAGA
1041  EKP
```

Supplementary Figure 1. Amino acid sequences of full-length myosin X and native myosin X HMM constructs used in this study. (**A**) M10^{Full}LZ. (**B**) BAP-M10$^{1–979}$HMM. The tags for purification, motif and domain structures were shown beneath the amino acid sequence. Note that BAP-M10$^{1–979}$HMM lacks myosin X tail domains and does not have the forced dimer motif at the C-terminus, although M10^{Full}LZ has a leucine zipper motif at the C-terminal side of the tail domains.

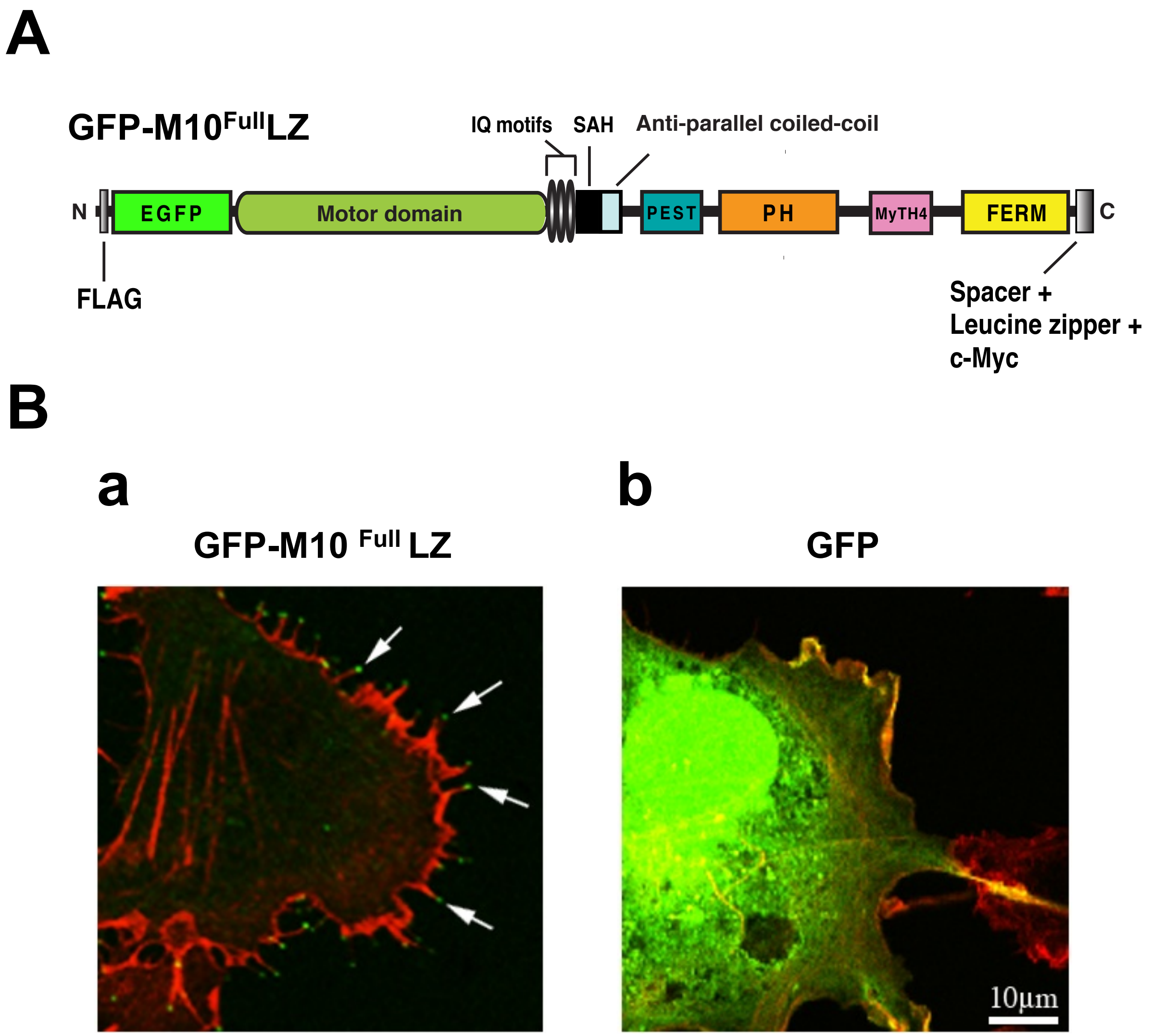

Supplementary Figure 2. Localization of GFP-full-length myosin X construct. (**A**) cartoon of domain structures of GFP-M10^{Full}LZ. GFP-M10^{Full}LZ consists of GFP, full-length myosin X$^{1-2052}$, followed by GCN4 leucine zipper motif. (**B**) ability of filopodia formation and localization of myosin X constructs in COS7 cells. (**a**) GFP-M10^{Full}LZ, (**b**) GFP. White arrows indicate GFP-M10^{Full}LZ localized at filopodial tips. Note that GFP-M10^{Full}LZ forms filopodia at cell peripheral regions, and localized at the tip of them. Scale bar: 10 mm for both in (**Ba**) and (**Bb**).

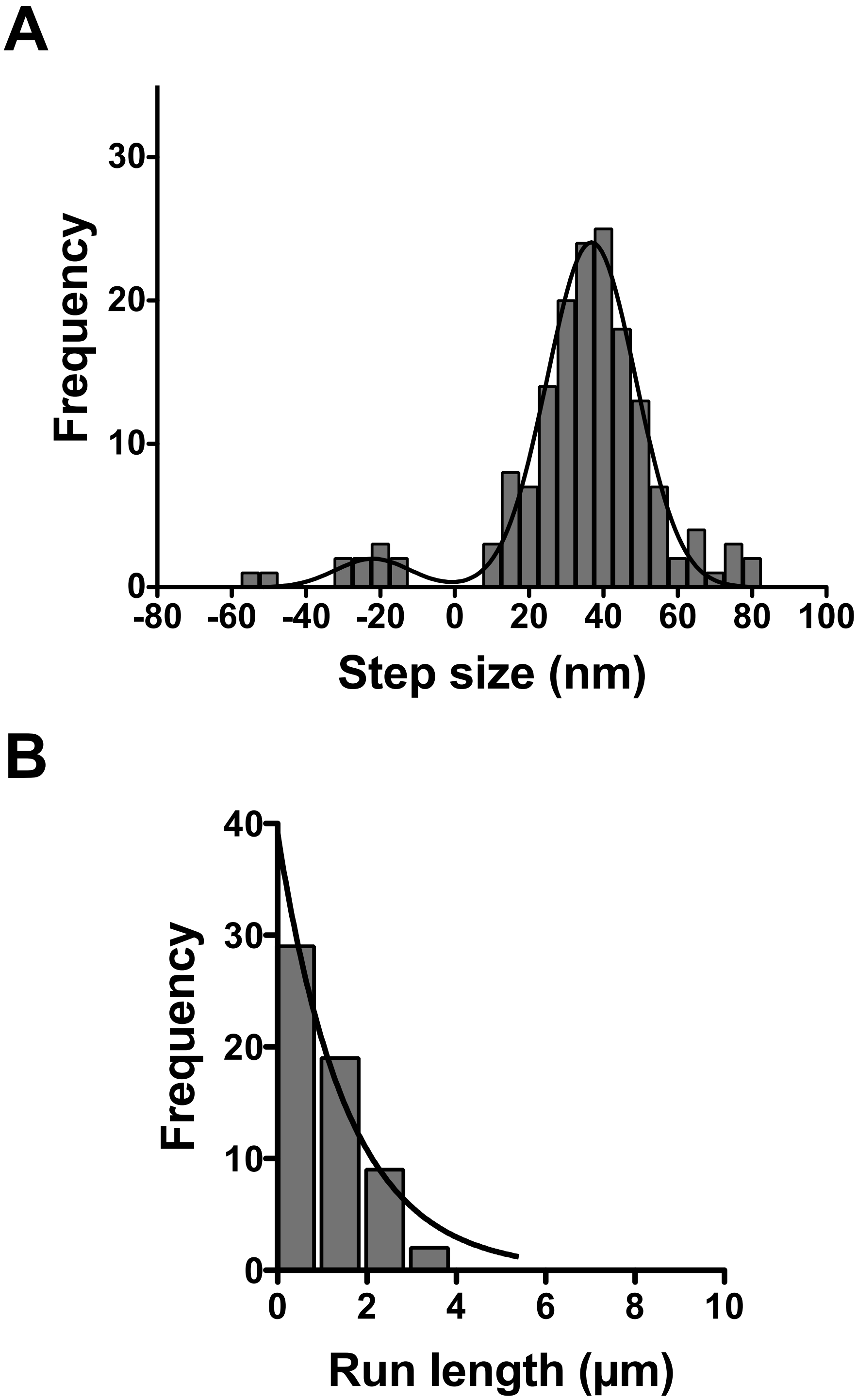

Supplementary Figure 3. Determination of step-size and run-length of myosin Va HMM as a control. The movement of mouse myosin Va HMM-Qdot655 was examined as described in Material and Methods. Experiments were done in the presence of 0.4 μM ATP, and the fluorescence images were captured at 50 fps. (**A**) step size distribution of myosin Va HMM. Black solid line shows the best fit to Gaussian equation. The mean step size of forward and backward steps are 36.8 ± 11.9 nm (mean ± s.d., n=151) and -22 ± 7.7 nm (s.d., n=11), respectively. (**B**) run length distribution of myosin Va HMM. Solid line shows the best fit to a single exponential equation, and the average run length was 1.6 ± 0.5 μm (s.e.m., n = 59).

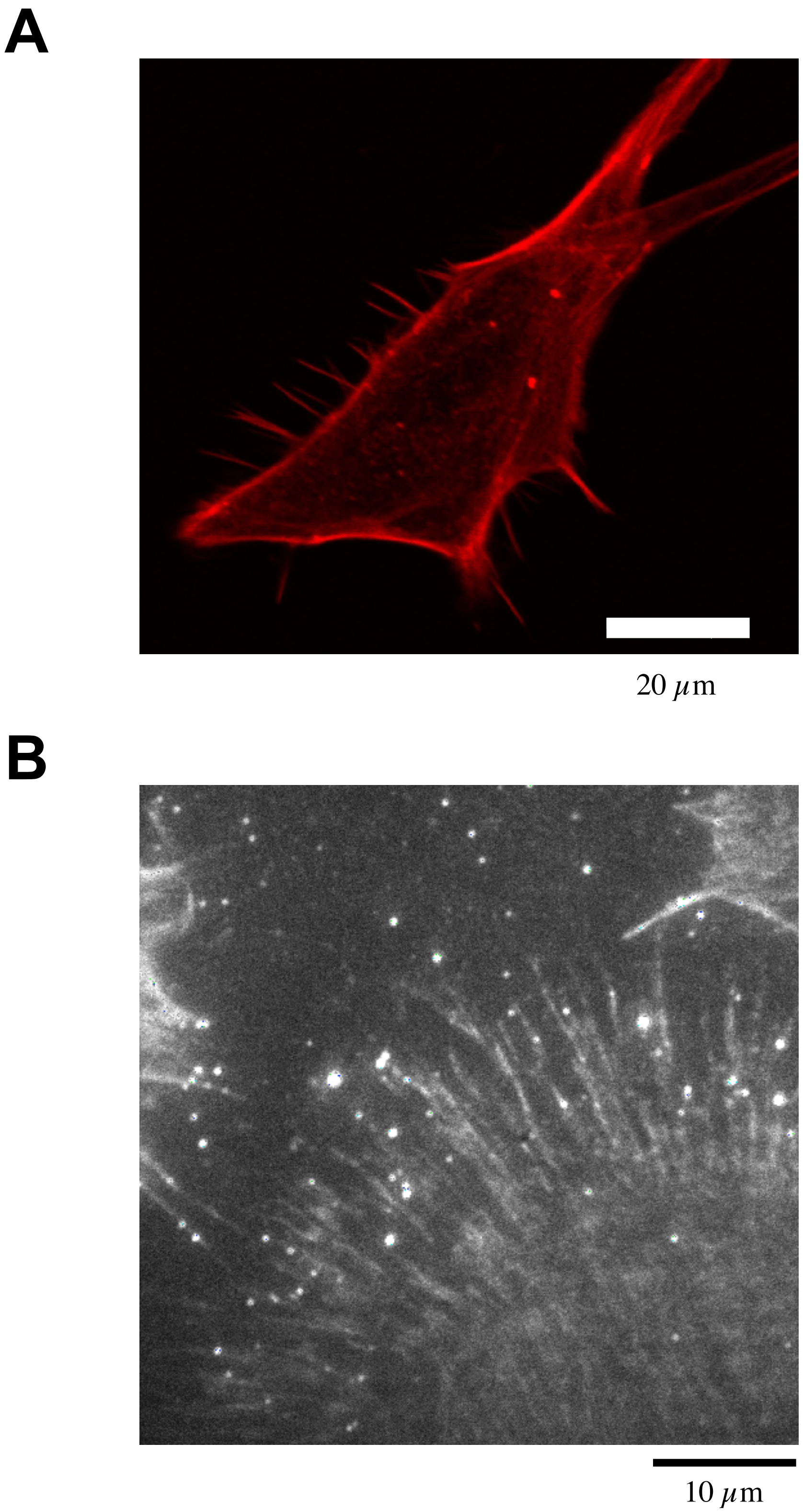

Supplementary Figure 4. Observation of M10^{Full}LZ-Qdots in permiabilized MEF3T3 cells. (**A**) permiabilized MEF3T3 cells stained with Alexa Fluor 568 phalloidin. The cells were observed with a laser scanning confocal microscope. (**B**) Localization of M10^{Full}LZ-Qdot655 on MEF3T3 cells. Note that M10^{Full}LZ-Qdots are observed on filopodia and around the cortical region of the cell. Scale bar: 20 μm for (**A**) and 10 μm for (**B**).

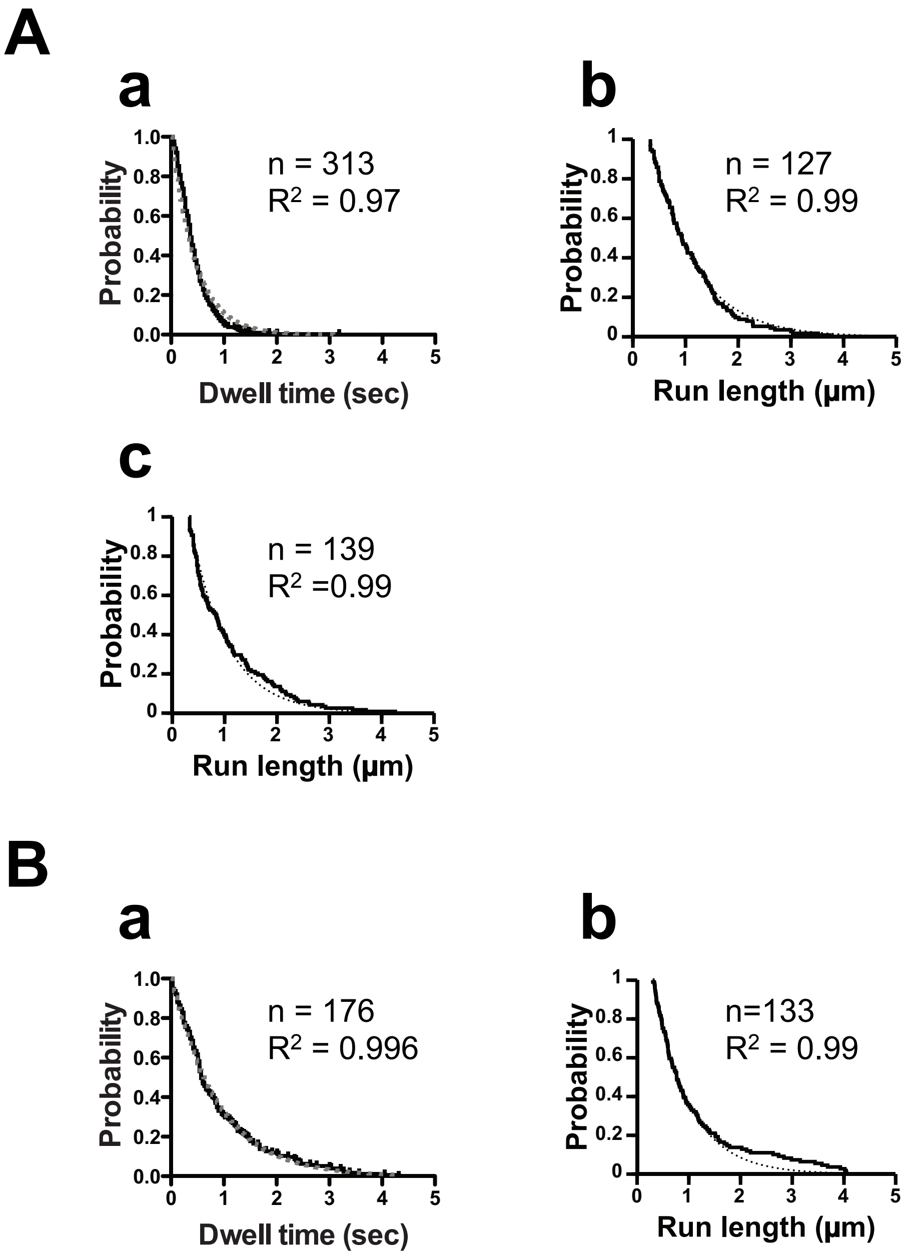

Supplementary Figure 5. Kaplan-Meier estimate of dwell time and run length survivor function. (**Aa**) dwell time on single actin filaments at 2 μM ATP. Dashed gray line shows a single exponential fit to the survival curve (the coefficient of determination $r^2 = 0.97$), giving a mean dwell time of 0.45 ± 0.01 s (s.e.m., n = 313). (**Ab**) run length on single actin filaments at 2 μM ATP. Events are left-truncated at 0.3 μm. Dashed line shows a single exponential fit to the survivor curve ($r^2 = 0.99$), yielding the run length decay constant of 0.82 ± 0.01 μm (s.e.m, n = 127). (**Ac**) run length on single actin filaments at 2 mM ATP (left-truncated at 0.3 μm). Dashed line shows a single exponential fit to the survival curve ($r^2 = 0.99$), yielding the run length decay constant of 0.70 ± 0.01 μm (s.e.m.). (**Ba**) dwell time on filopodia at 2 μM ATP. Dashed gray line shows a single exponential fit to the survival curve ($r^2 = 0.996$), giving a mean dwell time of 0.881 ± 0.007 s (s.e.m., n = 176). (**Bb**) run length in filopodia at 2 mM ATP (left-truncated at 0.3 μm). Dashed line shows a single exponential fit to the survival curve ($r^2 = 0.99$), giving the run length decay constant of 0.691 ± 0.006 μm (s.e.m., n = 133).

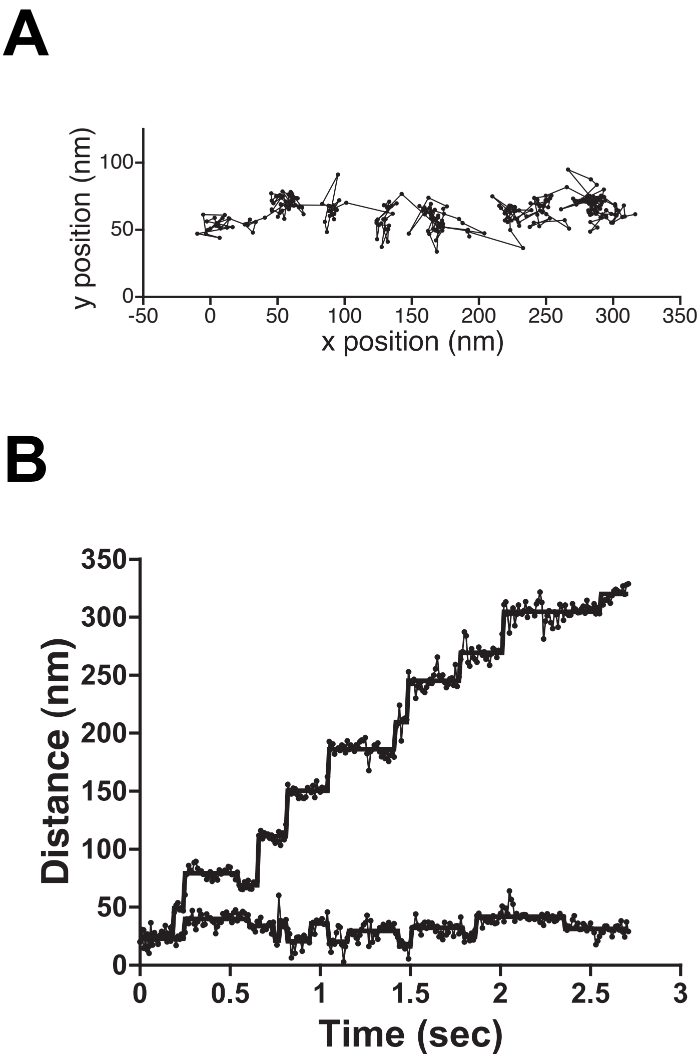

Supplementary Figure 6. Stepping of M10^{Full}LZ on filopodia under physiological ATP concentration. Experiments were done in the presence of 2 mM ATP, and the fluorescence images of M10^{Full}LZ-Qdot655 was captured at 100 fps. (**A**) an example of x-y position trace of M10^{Full}LZ stepping. (**B**) time versus distance representation. Upper and lower traces are the components from x and y positions, respectively. Solid lines represent the result of best fit with the step fitting software.

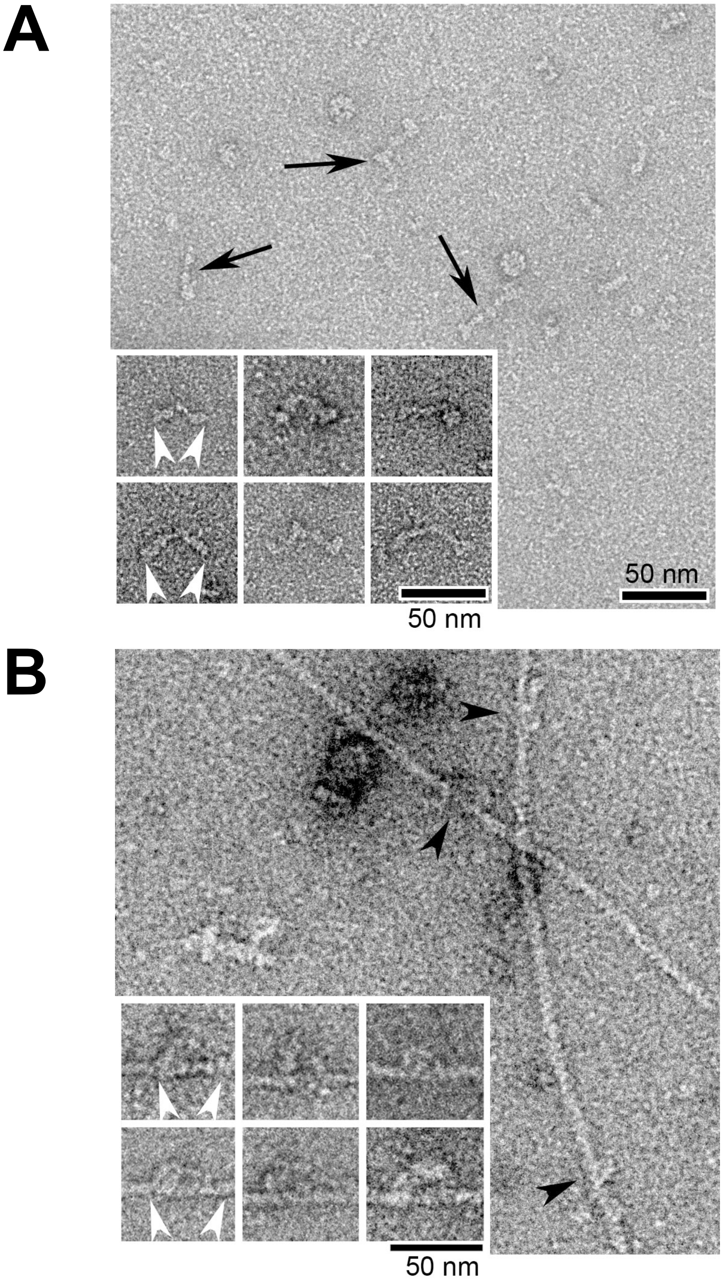

Supplementary Figure 7. Electron micrographic appearances of M10^{Full}LZ molecules and their binding to actin filament. (**A, B**) General appearances of M10^{Full}LZ molecules in the absence (**A**) and the presence of actin filaments (**B**). Black arrows and arrowheads point to two-headed molecules in each field. Inset images in (**A, B**) showed the galleries of individual molecules taken from each field: shorter and longer lengths of the inter-head conformers (top and bottom panels, respectively). White arrowheads in inset images indicate identified position of each head in inter-head conformers (**A**) and attaching positions of the heads along the actin filaments (**B**). The inter-head molecules clearly visualized attaching positions to the actin filaments were used for the length measurements. 50 nm scale bars in (**A**) applies to the fields and 50 nm scale bars in galleries (**A** and **B**) applies to inset images.

x-step (nm)	y-step (nm)
range	*mean ± s.e.m.*
10–30	26.4 ± 3.4 (n=20)
30–50	27.0 ± 2.6 (n=24)
50–70	33.1 ± 3.3 (n=6)
70–90	28.9 ± 6.4 (n=3)

Supplementary Table 1. Analysis of the diagonal M10 ^{Full}LZ movement. The absolute values of x-steps and the corresponding y-steps from the diagonal steps in Fig. 5 (I, II, III, and IV planes) were taken, and the relationship between x-step and y-step during diagonal step was analyzed. Note that the y-steps were relatively constant compared to the variance of the x-steps.

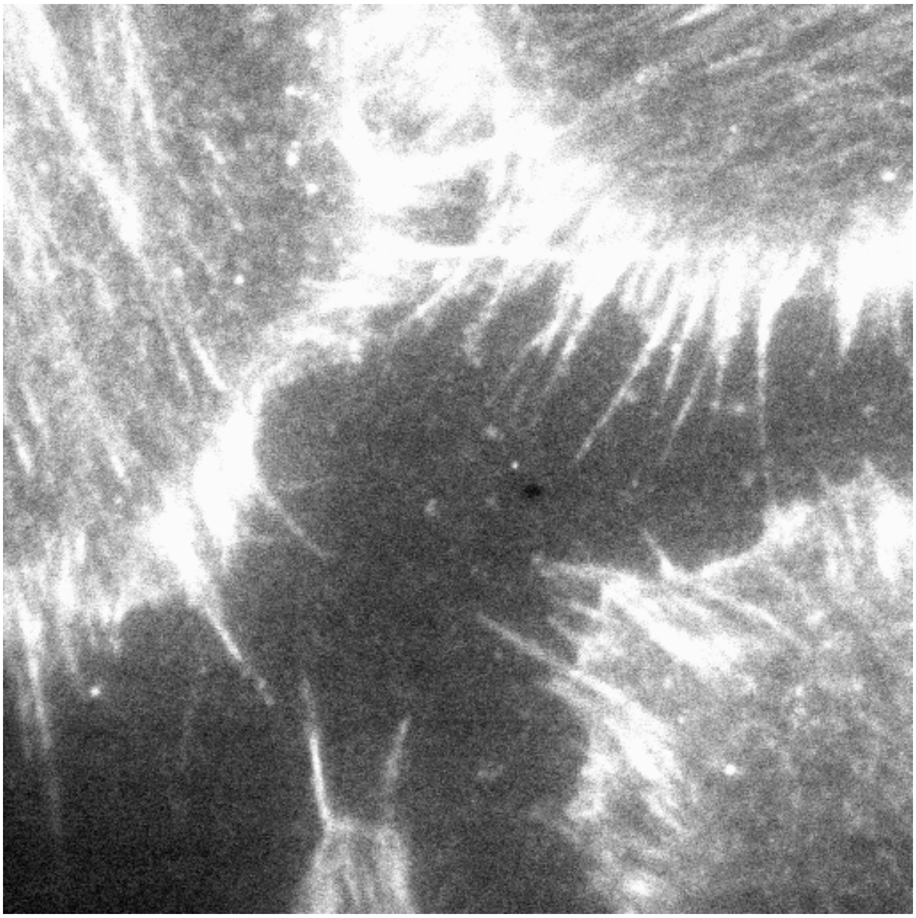

Legend to Supplementary Movie 1. Myosin X movement on filopodia. The movement of M10^{Full}LZ-Qdot at 2 μM ATP was captured at 33.3 fps and shorten the time 5 times using Adobe Premiere software. First 1 second show the image at the camera gain of 1000, followed by 200. M10^{Full}LZ-Qdot movement was observed mainly around filopodia.
